# Collateral benefits arising from mass administration of azithromycin in the control of active trachoma in resource limited settings

**DOI:** 10.11604/pamj.2014.19.256.3548

**Published:** 2014-11-07

**Authors:** Gabriel Kigen, Joseph Rotich, Jefitha Karimurio, Hillary Rono

**Affiliations:** 1 Department of Pharmacology and Toxicology, Moi University School of Medicine, Eldoret, Kenya; 2 Department of Epidemiology & Nutrition, Moi University School of Public Health, Eldoret, Kenya; 3 Department of Ophthalmology, University of Nairobi, Nairobi, Kenya; 4 Consultant Ophthalmologist, Kitale and Zonal eye surgeon, North Rift, Kenya

**Keywords:** Azithromycin, antibiotics, mass administration

## Abstract

**Introduction:**

The benefits of the use of antibiotics in the mass treatment for active trachoma and other diseases have been documented, but the secondary effects arising from such a programme have not been fully elucidated. The purpose of this study was to investigate the potential secondary benefits arising from the use of azithromycin in mass treatment of active trachoma in an economically challenged Kenyan nomadic community.

**Methods:**

Health information reports for January 2005 to December 2010 were reviewed to determine the annual trends of infectious diseases in the two districts, Narok and Transmara. The year 2007 was considered as the baseline for mass drug administration (MDA). Odds ratios (OR) were used to describe the association.

**Results:**

The mass distribution coverage in Narok was 83% in 2008, 74% in 2009 and 63% in 2010. The odds for malaria (OR = 1.13; 95% CI 1.12-1.14), diarrhoeal diseases (OR = 1.04; 95% CI 1.01-1.06), urinary tract infections (UTIs) (OR = 1.21; 95% CI 1.17-1.26), intestinal worms (OR, 4.98; 95% CI 4.68-5.3), and respiratory diseases other than pneumonia (OR, 1.15; 95% CI 1.13-1.16) were higher after three rounds of mass treatment, indicating a better outcome. Before the intervention, there was a reducing trend in the odds for respiratory diseases. In Transmara (control), there was an increase in odds for malaria, respiratory infections, UTIs and intestinal worms. The odds for diarrhoeal diseases, skin diseases and pneumonia decreased throughout the study period.

**Conclusion:**

Mass distribution of azithromycin may have contributed to the decrease in the prevalence of the respiratory infections in Narok District.

## Introduction

The mass administration of antibiotics has been used in the prevention and treatment of bacterial diseases. It has been recognized as a potentially vital and important public health strategy towards the control of neglected tropical diseases and other co-infections in sub-Saharan Africa, whereby the provision of universal healthcare is still a major challenge. It has also been considered as a cost-effective method because of geographic overlaps of various diseases and high level of poverty in the region. Despite this, the secondary health benefits effects of such a programme have not been fully expounded, especially in developing countries whereby provision of universal healthcare is still a major challenge. The purpose of this study was to investigate the secondary benefits arising from the mass administration of azithromycin, an antibiotic in the mass treatment of active trachoma in an economically challenged Kenyan nomadic community. This was achieved by examining the trends of diseases two years before, and after the drug administration. The administration of azithromycin was part of the SAFE strategy (Surgery, Antibiotics, Facial and Environmental cleanliness) conducted in Narok district in Kenya from 2007 to 2010. The F&E components had not been fully implemented in the entire district at the time of this study. The findings were compared with those of neighbouring Transmara district, which is trachoma-endemic but had no trachoma control project.

Trachoma is still endemic in developing countries and is the common cause of infectious blindness. Most of the afflicted are poor people with low hygiene and inadequate access to clean water. They are therefore vulnerable to several other infections [1, 2]. As a strategy to eradicate trachoma, the World Health Organization (WHO) adopted the SAFE programme consisting of Surgery, Antibiotics for infectious trachoma, Facial cleanliness to reduce transmission and Environmental cleanliness to improve hygiene and sanitation. It recommends mass distribution of antibiotic in affected areas with prevalence of active trachoma of more than 10% in children aged 1 to 9 years in a district, or 5% for the community [3, 4].

Trachoma control programs in some of districts are currently on going in Kenya. The SAFE programme, implemented in Shampole, Kajiado district reported reduction in prevalence of active trachoma from 46% to 16% and the potentially blinding trachoma from 4.5% to 1.7% over a period of one year with low incidences of recurrences [5]. SAFE strategy programme in the greater Narok district began in 2008 and proceeded annually for three years, with the support from Operation Eyesight Universal (OEU). The prevalence of trachoma in Narok district is 30.5% [6]. Azithromycin is currently the antibiotic of choice administered in repeated annual community mass treatment for a period of three years [7]. It is administered as a single dose of 1 gm for adults whereas the children's dose is 20 mg/kg. Expectant mothers and children less than six months are put on tetracycline eye ointment [8]. The F&E activities involve preventive measures to reduce disease transmission which includes the provision of water, health promotion and education. The full benefits of the F&E activities generally take a longer period to be realized owing to the challenges of its implementation [9–11].

Previous studies have demonstrated that azithromycin markedly decreases the prevalence of ocular strains of *Chlamydia trachomatis*, the causative agent for trachoma [12, 13]. Whereas mass treatment has been shown to reduce the prevalence of trachoma, the potential secondary health benefits of such treatment have not been well documented. Previous studies regarding its impact on the prevalence of respiratory infections have been inconclusive. In a study among children in Ethiopia, the authors reported a decline in the overall mortality, perhaps due to respiratory infections, diarrhoea and malaria [14, 15]. An indirect protective effect against trachoma among untreated children residing in villages where most individuals have been treated, and decline in pneumococcal resistance has also been reported after mass treatment in Ethiopia [16, 17]. However, other authors in related studies concluded that it may lead to the emergence of resistant strains of *Streptococcus pneumonia* [18, 19]. The aim of this study therefore, was to investigate the potential secondary benefits arising from the mass treatment.

## Methods

This community based operational research was conducted in the Narok County between August and November 2010. Permission to conduct the research was granted by the Institutional Research and Ethics Committee of Moi University. All the analyzed data were anonymized. Data on annual morbidity from January 2005 to December 2010 was obtained from Medical Officer of Health, Narok district. The data was collected using the standard Ministry of Health reporting tools from all health facilities. All patients attended to in these facilities were included in the study. Monthly records of the incidences of all new diseases and their frequencies in each facility were recorded. The monthly morbidity reports were then compiled and an annual summary determined. The prevalence of the seven most common infectious diseases was then assessed. We took 2007 as the baseline, since the implementation of the SAFE strategy commenced in 2008, and investigated the trends of the diseases two years before (2005 - 2006), and three years after (2008 - 2010). The Narok data for the period 2005 to 2007 (baseline) was used to establish the magnitude and “natural tends” (annual/monthly) of prevalent infectious diseases prior to MDA. Additionally, the 2007 to 2010 trend was compared with that for Transmara to determine whether the treatment was beneficial or not. At the time of the study, F&E activities in Narok were at its inception and had not achieved a wider coverage. In both Narok and Transmara districts, there were F&E activities being provided under the National Community Health Strategy programme. It was therefore assumed that any change in disease patterns was attributable to the MDA. We also reported the trends in the diseases.

### Statistical analysis

The number of patients treated in each month was aggregated to the totals for the year. The base year analysis was set to 2007, which was the year used to compute the test statistics. Analysis was performed using STATA software version.10 (STATACorp, College Station, Texas 7784) in order to compare the proportions of the various diseases before and after the intervention. Chi-square test was used to determine the association between respective years and the base year (2007) in respect of the various diseases. Odds ratios and significance of the chi-square test were used as indicators of prevalence. For years after 2007, an OR > 1 implied that the intervention had a beneficial effect, meaning that the proportion of cases in the following respective year was lower, or a decline in prevalence following the treatment/intervention. An OR < 1 implied that the intervention had no impact in the successive year (the proportion of the case in that year were significantly higher than the base year). The converse was true for the years before 2007. An (OR > 1) implied that the prevalence before the intervention was lower, that is the proportion of cases in 2007 was significantly higher than those in the prior year. An OR < 1 implied that the proportion was higher before the intervention.

## Results

**Figure 1:**
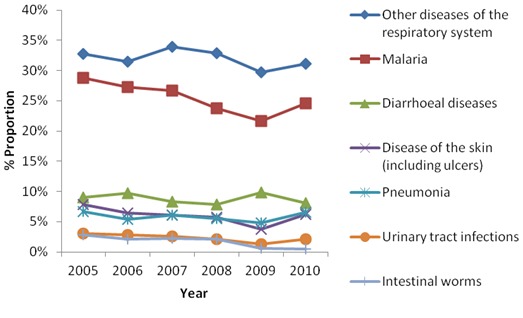
Comparison of the proportions of the patients with various illnesses over a period of five years in Narok district

The mass distribution coverage in Narok district decreased over the study period; 83% in 2008, 74% in 2009 and 63% in 2010. Respiratory diseases and malaria were the most prevalent diseases. The prevalence of pneumonia was between 5% and 7% while that of the other diseases of the respiratory system ranged from 30% to 34%. Malaria had a prevalence of between 22% and 29% over the study period. The prevalence of the other infections recorded is as in Table 1. Apart from the diarrhoeal diseases, there was a general decline in all the other recorded infectious diseases after the third year from the baseline, despite the decreased percentage coverage over the period (Table 1, Figure 1). Respiratory diseases were significantly more prevalent in the years prior to the intervention compared to the period after the use of antibiotics. The proportions of patients with malaria decreased throughout the study period. The prevalence for both diseases rose proportionately with the corresponding decrease in coverage from the second to the third round (malaria 25% vs. 22%, respiratory diseases 31% vs. 30%). There was a statistically significant increase in proportion of pneumonia during this period (Figure 1). There were statistically significant changes in the odds for all diseases except skin diseases three rounds after the intervention. The odds for malaria (68580(27%) vs. 63900(25%); OR, 1.13; 95% CI 1.12-1.14), diarrhoeal diseases (21253(8%) vs. 21010(8%); OR, 1.04; 95% CI 1.01-1.06), UTIs (6646(3%) vs. 5609(2%); OR, 1.21; 95% CI 1.17-1.26), intestinal worms (5834(2%) vs. 1218(0%); OR, 4.98; 95% CI 4.68-5.3), and respiratory diseases other than pneumonia (87163 (34%) vs. 81101 (31%); OR, 1.15; 95% CI 1.13-1.16) were higher after three rounds of mass treatment, indicating a better outcome or a reduction in the prevalence of the diseases. The odds for pneumonia decreased (15742(6%) vs. 17001(7%); OR, 0.94; 95% CI 0.92-0.96), although the odds were higher after the second round 15742(6%) vs. 9750(5%); 1.32(1.28-1.35) before rising after the third round. There was no significant change in odds for the skin diseases.

**Table 1:** Proportion of patients with various illnesses at the baseline and after mass administration of azithromycin in Narok District

	Number of patients (%)									
Year	*n*_*1*_ (2007/08)	2007* *n*_*2*_ (%)	2008 *n*_*2*_ (%)	2008 vs. 2007	*n*_*1*_ (07/09)	2009 *n*_*2*_ (%)	2009 vs. 2007	*n*_*1*_ (07/10)	2010	2010 vs. 2007
Condition or symptom	Total no. of patients	*n*_*2*_ = 255132	124472 (%)	OR (95% CI)	Total no. of patients	*n*_*2*_ =204792	OR (95% CI)	Total no. of patients	*n*_*2*_ = 260359	OR (95% CI)
Pneumonia	23310	15742(6)	7568(6)	1.02(0.99-1.05)	25492	9750 (5)	1.32(1.28-1.35)	32743	17001(7)	0.94(0.92-0.96)
Other diseases of the respiratory system	133041	87163(34)	45878(37)	0.89(0.88-0.90)	148123	60960(30)	1.22(1.21-1.24)	168264	81101(31)	1.15(1.13-1.16)
Diarrhoeal diseases	31759	21253(8)	10506(8)	0.99(0.96-1.01)	41349	20096(10)	0.84(0.82-0.85)	42263	21010(8)	1.04(1.01-1.06)
Malaria	101156	68580(27)	32576(26)	1.04(1.02-1.05)	112965	44385(22)	1.33 (1.31-1.35)	132480	63900(25)	1.13(1.12-1.14)
Intestinal worms	8287	5834(2)	2903(2)	0.98(0.94-1.03)	7141	1307 (1)	3.64(3.43-3.87)	7052	1218(0)	4.98(4.68-5.3)
Disease of the skin (including ulcers)	23371	15519(6)	7852(6)	0.96(0.94-0.99)	23233	7714 (5)	1.65(1.61-1.70)	31513	15994(6)	0.99(0.97-1.01)
Urinary tract infections	9663	6646(3)	3017(2)	1.08(1.03-1.12)	9371	2725 (1)	1.98(1.90-2.07)	12255	5609(2)	1.21(1.17-1.26)

*n_1_* = Total number of patients with a certain condition for the year and the baseline (2007*) e.g. *n_1_* for pneumonia in 2007/2008 = 23310 (that is the sum of 15742 and 7568)

*n_2_* = Total number of patients with all conditions for the respective year, e.g. the number of patients with pneumonia in 2007 = 15742 which is 6% of *n_2_*

**Figure 2:**
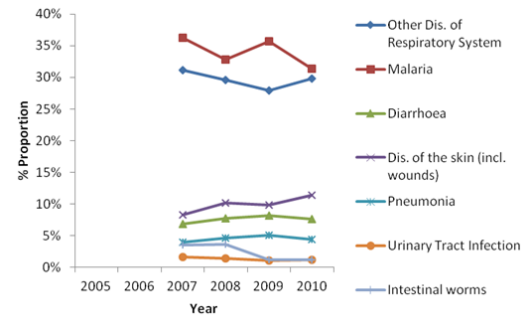
Comparison of the proportions of the patients with various illnesses between 2007 and 2010 in Transmara district

In Transmara (control), there was an increase in the odds for malaria, respiratory infections, UTIs and intestinal worms. The odds for diarrhoeal diseases, skin diseases and pneumonia decreased but significantly in this case, the odds decreased throughout the period (Table 3, Figure 2). There were no records for Transmara for the 2005 and 2006 years. The crossing of the residents from Transmara to Narok district may have affected the results. The number of patients in the study was quite high and therefore any small percentage change translated in statistically significant changes (Table 1, Table 2). There were no records for Transmara for the 2005 and 2006 years. The crossing of the residents from Transmara to Narok district may have affected the results. The number of patients in the study was quite high and therefore any small percentage change translated in statistically significant changes (Table 1, Table 2). There was a change from manual to electronic data recording in 2008 which could have resulted in the loss of some data. This may explain the comparatively low numbers recorded in 2008.

**Table 2:** Proportion of patients with various illnesses before the mass administration of azithromycin in Narok District

	Number of patients (%)						
Year	*n*_*1*_ (05/07)	2005 *n*_*2*_ (%)	2005 vs. 2007	*n*_*1*_ (06/07)	2006*n*_*2*_ (%)	2006 vs. 2007	2007* *n*_*2*_ (%)
Condition or symptom	Total no. of patients	*n*_*2*_ = 208221	OR (95% CI)	Total no. of patients	*n*_*2*_= 224954	OR (95% CI)	*n*_*2*_ = 255132
Pneumonia	29665	13923(7)	0.92 (0.9-0.94)	27894	12152(5)	1.15 (1.02-1.07)	15742(6)
Other diseases of the respiratory system	155484	68321(33)	1.06 (1.05-1.08)	158846	71683(32)	1.11 (1.1-1.12)	87163(34)
Diarrhoeal diseases	39933	18680(9)	0.92 (0.9-0.84)	43313	22060(10)	0.84 (0.82 -0.85)	21253(8)
Malaria	128511	59931(29)	0.91(0.9-0.92)	130482	61902(28)	0.97 (0.96-0.98)	68580(27)
Intestinal worms	11597	5763(3)	0.82(0.79-0.85)	10520	4686(2)	1.10 (1.06-1.14)	5834(2)
Disease of the skin (including ulcers)	31753	16234(8)	0.77 (0.75-0.78)	30479	14690(7)	0.93 (0.91-0.95)	15519(6)
Urinary tract infections	13077	6431(3)	0.84 (0.81-0.87)	13027	6381(3)	0.92 (0.88-0.95)	6646(3)

**Table 3:** Transmara, Proportion of patients with various illnesses at the baseline and one and two years after the mass administration of azithromycin in Transmara District

	Number of patients (%)									
Year	*n*_*1*_ (07/08)	2007* *n*_*2*_ (%)	2008 *n*_*2*_ (%)	2008 vs. 2007	*n*_*1*_ (07/09)	2009 *n*_*2*_ (%)	2009 vs. 2007	*n*_*1*_ (07/010)	2010 *n*_*2*_ (%)	2010 vs. 2007
Condition or symptom	Total no. of patients	*n*_*2*_ = 156839	*n* = 141628	OR (95% CI)	Total no. of patients	*n*_*2*_= 199599	OR (95% CI)	Total no. of patients	*n*_*2*_= 233313	OR (95% CI)
Pneumonia	12683	6170(4)	6513(5)	0.85(0.82-0.88)	16408	10238(5)	0.76(0.73-0.78	16437	10267(4)	0.89(0.86-0.92)
Other diseases of the respiratory system	90853	48898(31)	41955(30)	1.08(1.06-1.09)	104526	55628(28)	1.17(1.16-1.19)	118442	69544(30)	1.07(1.05-1.08)
Diarrhoeal diseases	21772	10789(7)	10983(8)	0.88(0.85-0.9)	27022	16233(8)	0.83(0.81-0.86)	28518	17729(8)	0.9(0.88-0.92)
Malaria	103311	56856(36)	46455(33)	1.17(1.15-1.18)	59246	2390(1)	46.92(45.01-48.92)	129932	73076(31)	1.25(1.23-1.26)
Intestinal worms	10718	5614(4)	5104(4)	0.99(0.96-1.03)	10300	4686(2)	1.54(1.48-1.61)	8347	2733(1)	3.13(2.99-3.28)
Disease of the skin (including ulcers)	27385	12970(8)	14415(10)	0.80(0.78-0.82)	32630	19660(10)	0.83(0.81-0.84)	39494	26524(11)	0.7(0.69-0.72)
Urinary tract infections	4432	2488(2)	1944(1)	1.16(1.09-1.23)	4562	2074(1)	1.54(1.45-1.63)	5372	2884(1)	1.29(1.22-1.36)

## Discussion

The provision of basic healthcare is still a major challenge in developing countries and respiratory diseases, malaria and diarrhoea have been identified as the leading death of children in developing countries [20–22]. Mass drug administration and integrated control has now been recognized as a vital and important public health solution towards the control of neglected tropical diseases and other co-infections in sub-Saharan Africa [23–25]. Previous studies have demonstrated that this strategy is a cost-effective method because of the geographic overlaps of the various diseases and the high level of poverty in the region. It has also been shown to be safe and efficacious [26, 27]. Our study was set in a community that is poor and of low hygiene, and therefore quite vulnerable to several diseases due to inaccessibility of water and sanitation among other problems. The provision of health services is also a major challenge owing to their pastoralist and nomadic way of life. The purpose of our study was to investigate the potential benefits arising from the mass administration of azithromycin in resource-limiting settings. We utilized the data from an existing programme for the control of infectious trachoma to determine the potential secondary effects resulting from mass administration of azithromycin. Our results suggest that the mass administration of azithromycin may have some secondary beneficial effects.

Antibiotic prophylaxis has been used to reduce the incidences of streptococcal infections, and azithromycin has been shown to control pneumonia epidemics in army recruits [28–31]. In addition, azithromycin and chloroquine combination has been shown to be effective in the prevention and treatment of both malaria and sexually transmitted diseases in pregnancy [32, 33]. The collateral benefits of antibiotic prophylaxis in Africa have not been well documented. Most of the reported studies relate to the use of azithromycin in trachoma control programs. The authors in previous studies conducted in rural Ethiopian villages concluded that the mass administration of azithromycin led to a decline in the respiratory infections and pneumococcal resistance. In addition there was a reported decrease in the prevalence of malaria and diarrhoea [14, 17]. In a separate study conducted in Gambia, the authors concluded that it reduced the childhood morbidity [34]. A recent study, also conducted in Ethiopia concluded that mass administration of azithromycin in a trachoma control programme reduced all-cause and infections childhood mortality [15]. We observed a decrease in the prevalence of respiratory infections, including pneumonia upon intervention with azithromycin.

Malaria is only second to HIV/AIDS as a cause of death in Sub-Saharan Africa, and is the leading cause of death in children below the age of five whereby it is responsible for over 800,000 million deaths each year [22, 35–37]. From our studies, there was a general decline in the proportion of patients with malaria throughout the study period with a sharp decline two years after the intervention. This could in part be attributed to an ongoing parallel programme in the whole region (Narok and Transmara included), which involved the distribution of mosquito of nets. However it should be noted that azithromycin has been demonstrated to posses the potential for prophylaxis and treatment of malaria alone, or in combination of other therapies [38–41]. Indeed it has been tried in Africa, and in a trial in Western Kenya, the authors concluded that it was efficacious for prophylaxis against malaria [42, 43]. Studies conducted in Asia have also demonstrated that azithromycin is quite tolerable in the treatment of malaria [44].

The mass distribution coverage of the population in Narok district was 83% in 2008, 74% in 2009 and 63% in 2010 respectively [6]. Our findings indicated an increase in the incidences of diarrhoeal diseases after the second year. These results are inconsistent with earlier findings conducted in Nepal, Ethiopia and Gambia respectively [14, 18, 34]. This could be attributed to several other factors including hygiene and sanitation, as well as the weather patterns. There were significant decreases in the diseases of the skin, urinary tract infections and reported cases of intestinal worms after the second year. Azithromycin has been shown to reduce the incidents of STIs in previous studies conducted on commercial sex workers in Philippines [45].

These findings suggest that the mass distribution appears to reduce incidence of the infectious diseases. Antibiotic prophylaxis is justifiable in the management of trachoma and several other diseases. However careful considerations have to be made with regards to the benefits of mass use of antibiotics. The literature is awash with specific dangers of non-specific mass use of antibiotics such as development of resistance and adverse drug effects among others. Continuous surveillance is important to assess the actual benefits of such a programme. But it is also important to consider the cost-benefit ratio especially in such settings where the residents have little access to health services owing to poverty, illiteracy and their way of life. Further studies should be conducted to ascertain the adverse and beneficial effects of such approaches in the development of public health policies in such settings.

There are several limitations in our study. We did not obtain data on the demographics of the patients and were therefore unable to determine the extent any confounding factors to the results. Some patients may not have been seen after the administration of azithromycin. We also made the assumption that the diagnosis was correct. Most of the previous studies have been undertaken in children less than five years, whereby antibiotic administration may make better impact owing to the less developed immunities. We could only obtain records for the years 2008-2010. There are also several other confounders which include the weather, which may have affected the disease patterns of diarrhoea, pneumonia and malaria. The migration of the residents in response to the weather changes and the difficulties in measuring the effects of F&E could have also affected our results.

## Conclusion

Our findings demonstrate that the mass distribution of azithromycin may have contributed to the decrease in the prevalence of the respiratory infections in Narok District.
